# Frontiers of Ultrasound Technology in Prostate Cancer Treatment

**DOI:** 10.1111/iju.70160

**Published:** 2025-06-22

**Authors:** Sunao Shoji, Kumpei Takahashi, Jun Naruse, Yoshiaki Kawamura

**Affiliations:** ^1^ Department of Urology Tokai University School of Medicine Isehara Japan

**Keywords:** clinical outcomes, high‐intensity direction ultrasound, high‐intensity focused ultrasound, low‐intensity focused ultrasound, prostate cancer

## Abstract

Focal therapy is a minimally invasive alternative treatment for localized prostate cancer (PC) aiming to achieve oncological control while preserving urinary and sexual functions. High‐intensity focused ultrasound (HIFU) and high‐intensity directional ultrasound (HIDU) enable precise prostate ablation guided by real‐time transrectal ultrasound or magnetic resonance imaging (MRI) fusion imaging. This review evaluates the current status and prospects of ultrasound technology in the treatment of PC. HIFU provides precise tumor ablation with minimal damage to the surrounding tissues, whereas HIDU allows MRI‐guided transurethral treatment with real‐time thermometry. Clinical studies have reported favorable biochemical recurrence‐free survival and functional outcomes. In Japan, a multi‐institutional prospective study on focal therapy using HIFU has been approved as an advanced medical technology by the Minister of Health, Labor, and Welfare. This study compared the oncological and functional outcomes of HIFU‐based focal therapy and radical prostatectomy in pair‐matched patients to evaluate the effectiveness of HIFU in treating localized PC. HIFU induces immunogenic cell death, releases tumor‐associated antigens, and reduces immunosuppressive cells. Low‐intensity focused ultrasound enhances tumor immunogenicity by promoting heat shock protein expression and CD8^+^ T cell activation. In conclusion, HIFU and HIDU offer effective focal therapy options for localized PC, and the immunomodulatory properties of HIFU and low‐intensity focused ultrasound may benefit advanced PC treatment. Future studies should optimize patient selection by developing advanced imaging technologies and predictive models capable of visualizing MRI‐invisible lesions and accurately detecting micro‐metastatic disease. Moreover, evaluating long‐term outcomes and exploring their combination with immunotherapy to enhance oncological efficacy will be essential.

**Abbreviations**
AS: active surveillance
BFSRs: biochemical‐free survival rates
csPC: clinically significant PC
EPIC: Expanded Prostate Cancer Index Composite
FFS: failure‐free survival
HIDU: high‐intensity directional ultrasound
HIFU: high‐intensity focused ultrasound
IIEF‐5: International Index of Erectile Function‐5
LOFU: low‐intensity focused ultrasound
mpMRI: multi‐parametric MRI
MRI: magnetic resonance imaging
NHT: neoadjuvant hormone therapy
OS: overall survival
PC: prostate cancer
PSA: prostate specific antigen
RARP: robot‐assisted radical prostatectomy
TRUS: transrectal ultrasound

## 1 Introduction

The standard management of localized prostate cancer (PC) includes active surveillance (AS) and whole‐gland treatment. AS is the preferred disease management strategy for selected patients with low‐ and intermediate‐risk localized PC [1]. In contrast, whole‐gland treatment, such as radical prostatectomy or radiation therapy, is recommended for patients with localized PC with an estimated life expectancy of > 10 years without the presence of small cancerous lesions and a Gleason score of 3 + 3 [2]. However, the deterioration of urinary [3] and sexual functions [4] is considered to be the main issue in post‐radical prostatectomy. Radiation‐induced hemorrhagic cystitis [5] and severe rectal bleeding [6] are risk factors for mortality after radiation therapy.

Recently, ultrasound treatments, such as transrectal high‐intensity focused ultrasound (HIFU) [7] and high‐intensity directional ultrasound (HIDU) [8] have attracted attention as modalities for focal therapy in localized PC. However, HIFU is a promising treatment for localized PC, supported by its favorable long‐ and medium‐term clinical outcomes. It is widely used because it allows precise millimeter‐level treatment planning in real time using transrectal ultrasound (TRUS) [9, 10] and magnetic resonance imaging (MRI) [11, 12], allowing for a distinct margin between the treated area and the adjacent untreated tissue without puncturing or cutting the prostate [13]. As no special construction work is required for installation, TRUS‐guided HIFU is widely used as an ultrasound treatment device for localized PC, particularly for focal therapy [7]. In HIDU, a continuously sweeping directional ultrasound beam is delivered from the prostatic urethra based on real‐time MRI for planning, thermometry, and cooling of the urethra and rectum [8]. Ultrasound treatment is expected to attract increasing attention as a treatment strategy for PC because it allows seamless integration of PC localization diagnosis via medical imaging into treatment planning. Additionally, its potential immunostimulatory effects have recently attracted attention.

This review summarizes the frontiers of ultrasound technology in the treatment of PC.

## 2 Principles and Devices for Ultrasound Treatment of PC

**FIGURE 1:**
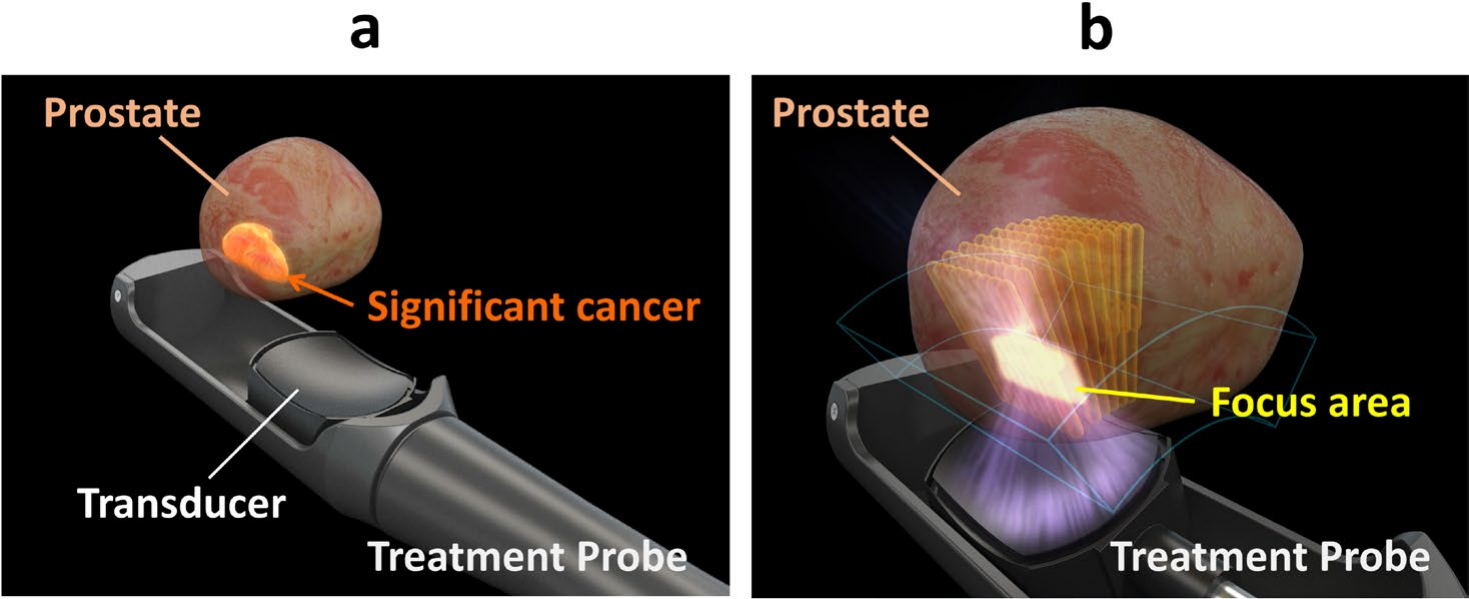
Computer graphic images of a commercial device for high‐intensity focused ultrasound for prostate cancer, Sonablate 500. In high‐intensity focused ultrasound (HIFU) treatment, a probe is inserted into the rectum, and the treatment area is planned using transrectal ultrasound imaging (a), which then emits ultrasound waves from a spherical transducer, delivering focused energy to millimeter‐sized target points, where its thermal and mechanical effects destroy the prostate tissue, with the focal areas moving in an overlapping pattern to cover the entire planned region (b). The image usage has been approved by Sonablate Corp.

**FIGURE 2:**
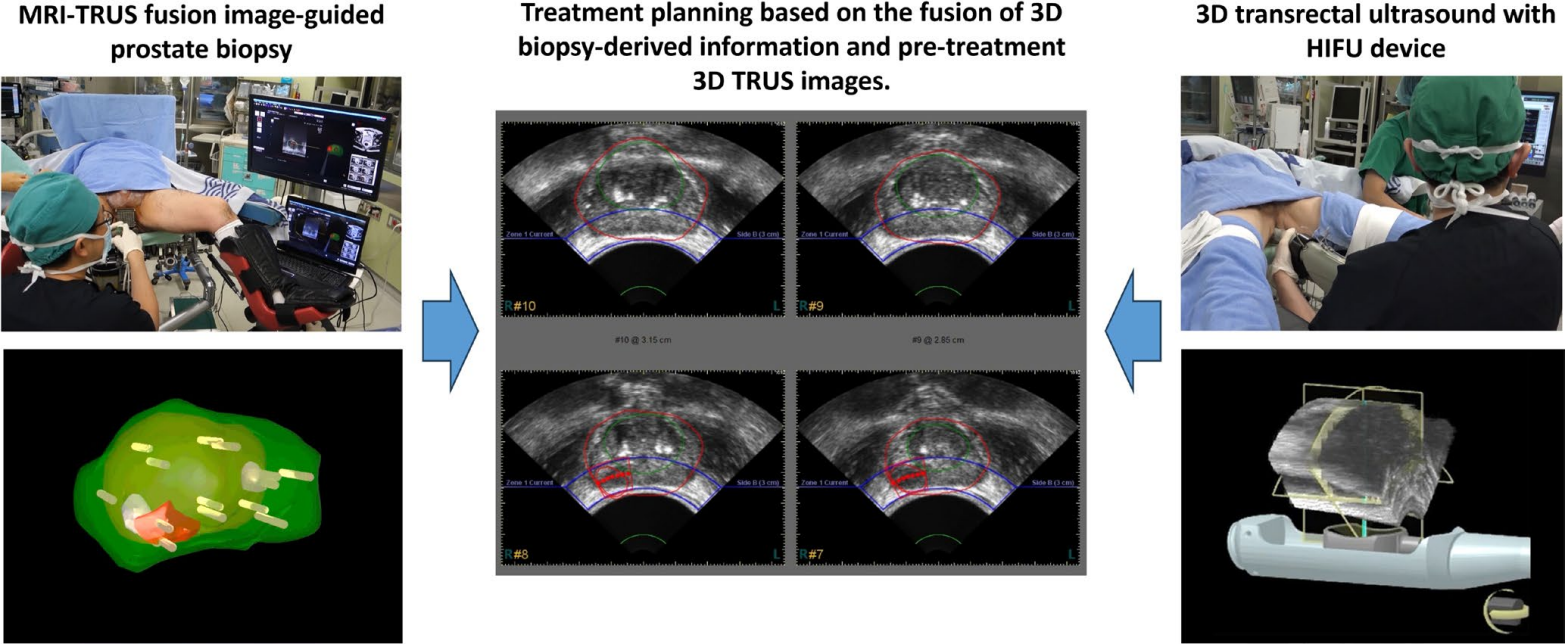
Magnetic resonance imaging‐transrectal ultrasound (TRUS) fusion image‐guided treatment planning in focal therapy using high‐intensity focused ultrasound. In commercial high‐intensity focused ultrasound (HIFU) devices for prostate cancer (Sonablate), treatment planning utilizes high‐resolution TRUS images. To achieve precise localization of the treatment area, three‐dimensional (3D) cancer localization data from preoperative MRI‐TRUS fusion image‐guided biopsies were integrated with 3D ultrasound images from the HIFU device. This fusion enables the visualization of the target area in a two‐dimensional format, assisting in accurate and effective treatment planning.

**FIGURE 3:**
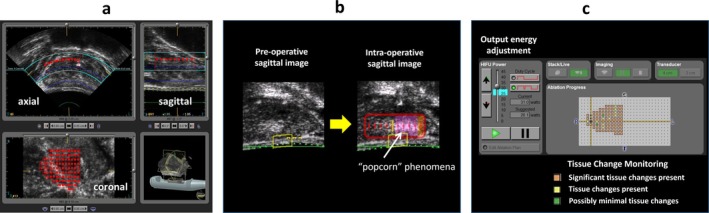
Treatment planning and real‐time monitoring in HIFU. (a) Cross‐sectional treatment planning: High‐intensity focused ultrasound (HIFU) treatment planning is performed using three cross‐sectional views of the prostate: Axial, sagittal, and coronal. These views enable precise localization of the treatment area, ensuring accurate energy delivery to the targeted region. (b) The “popcorn” phenomenon refers to the appearance of hyperechoic areas within the treated region on B‐mode ultrasound imaging during HIFU therapy. This phenomenon is caused by tissue cavitation during treatment and serves as an indicator that sufficient energy has been delivered to the targeted area, confirming that effective ablation has been achieved. (c) Real‐time energy adjustment and tissue monitoring: The Sonablate device allows for real‐time TRUS imaging, enabling energy adjustments based on the appearance of cavitation phenomena and continuous tissue change monitoring, ensuring optimal treatment precision.

HIFU produces ultrasound waves generated by a spherical transducer, delivering ultrasonic energy to a focus area, which are pinpoint foci of millimeters in diameter [14]. The thermal and mechanical effects of HIFU destroy the target prostate tissue [14] (Figure 1), and the anti‐cancer effect of HIFU has been evaluated in rats with subcutaneously implanted PC cell lines [15, 16]. In a study on a canine prostate model for clinical application, coagulative necrotic changes were found in a focally ablated area with distinct margins after a 1‐s exposure with an acoustic intensity of 1000 W/cm [2, 17]. Minimal changes in tissues surrounding the area ablated with HIFU have also been reported [18]. Madersbacher et al. studied focal ablation using HIFU in 10 patients before radical prostatectomy. They measured the temperature in the treated area with a trans‐peritoneal thermocouple and found a range of 70°C–98.6°C during ablation. The results confirmed that the targeted prostate area was accurately treated [13]. Additionally, Beerlage et al. verified that HIFU successfully caused focal ablation and necrosis by examining prostate specimens removed immediately after hemi‐ablation [19]. PC treatment planning using commercial HIFU devices is based on high‐resolution TRUS imaging. To accurately define the treatment area, three‐dimensional (3D) cancer localization data from preoperative MRI‐TRUS fusion image‐guided biopsies were combined with 3D ultrasound images from the HIFU device. This integration allowed the target area to be displayed as a two‐dimensional (2D) image (Figure 2). Treatment planning was performed using three cross‐sectional views of the prostate: axial, sagittal, and coronal (Figure 3a). During treatment, the “popcorn” phenomenon, caused by tissue cavitation, indicates that the targeted area has received sufficient energy for effective ablation (Figure 3b) [20]. The energy can be adjusted using real‐time TRUS images to determine the appearance of the phenomena [21] and tissue change monitoring in the Sonablate device (Figure 3c). HIFU devices include several safety features such as rectal cooling systems, automatic rectal wall position monitoring, and reflectivity index systems. The endorectal probe used for HIFU is covered with a protective sheath, inserted into the rectum, and filled with cooling water or fluid. This cooling system continuously circulates liquid to keep the rectal wall temperature below 22°C, thereby preventing heat damage during treatment [22]. An automatic rectal wall position system continuously measures the distance between the probe and the rectal wall, helping to prevent unintended treatment outside the target area. Additionally, the reflectivity index system detects changes in the rectal wall on TRUS images and alerts the operator during treatment to ensure safe and accurate ablation [22].

**FIGURE 4:**
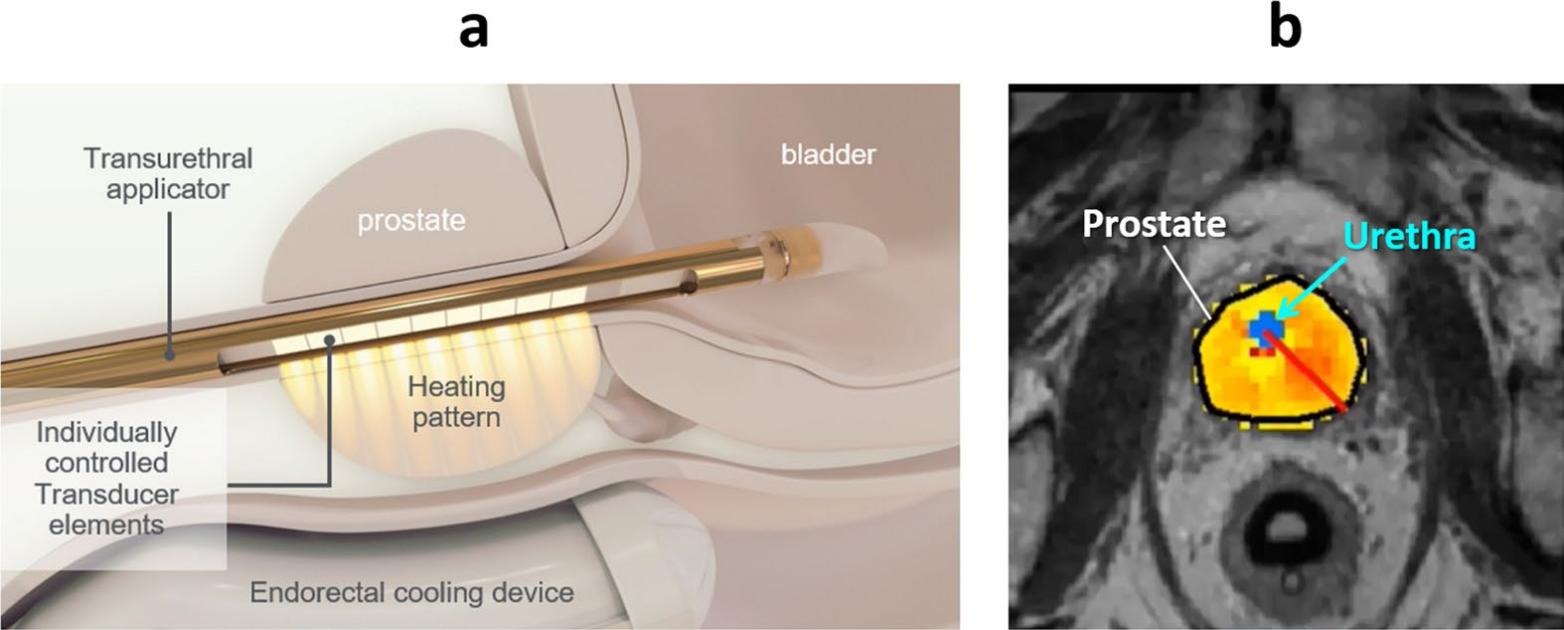
Schema of transurethral high‐intensity directional ultrasound for localized prostate cancer, TULSA‐PRO. (a) For the treatment of transurethral high‐intensity directional ultrasound (HIDU) for localized prostate cancer, the transurethral applicator, which includes individually controlled transducer, is inserted. (b) During treatment, the HIDU transducer rolls and treats the planned prostate area based on the thermometry on magnetic resonance imaging. The image usage has been approved by Profound Medical.

Recently, MRI‐guided ultrasound therapy has been introduced for the treatment of localized PC. This technique provides thermal feedback and real‐time power adjustment to ensure optimal tissue ablation temperature. In MRI‐guided HIFU, the endorectal probe is covered with a protective sheath and filled with degassed water at 14°C to cool and protect the rectum during treatment. T2‐weighted MRI in three planes and diffusion‐weighted imaging are used for precise treatment planning [23]. MRI‐detectable lesions with a 5‐mm safety margin, including the anterior rectal wall, are targeted using the macrosonication technique that applies ultrasound to multiple focal points [23]. During treatment, real‐time MRI thermography monitors both the target area and the rectum [23]. The procedure is considered successful if the target temperature exceeds 65°C [23]. HIDU for PC was designed as an MRI‐guided transurethral ultrasound ablation for monitoring intraprostatic temperature using MRI thermometry (Figure 4) [24]. In a canine study, this treatment successfully generated precise and controlled heating in the targeted area of the prostate [24]. In this study, the acoustic power was adjusted to maintain a temperature of 55°C at the outer edge of the target area, based on temperature measurements taken every 5 s using magnetic resonance thermometry. Tissue analysis by hematoxylin and eosin staining confirmed that the ablated area had undergone thermal damage [24]. Chopra et al. conducted a study on HIDU treatment for localized PC in eight patients before radical prostatectomy [25], and observed high precision, with a temperature variation of < 2°C and a targeting accuracy of −1.0 ± 2.6 mm [25]. The average temperature at the edge of the treated area was 52.3°C ± 2.1°C, and the treatment rate was 0.5 mL/min [25].

HIFU and HIDU have similar limitations in patients with large calcifications because large calcifications prevent ultrasound penetration. HIFU and HIDU are unable to treat PC located in the transition and peripheral zones in patients who have large calcifications without transurethral resection of the prostate (TUR‐P) to remove the calcification. Another limitation is related to the penetration depth of ultrasound. Because the penetration depth of HIFU is limited, the anterior portion of a large prostate cannot be completely treated and requires size reduction with TUR‐P or hormonal therapy before treatment. Similarly, the energy reaching the limbus of the large prostate decreased in HIDU. When TUR‐P is performed, some prostate tissues are resected along with the prostate calculi. In HIFU treatment, the TUR cavity created by TUR‐P did not significantly affect the procedure. However, in HIDU, the energy output is automatically adjusted based on the distance between the urethra and prostate margin. Because ultrasound travels more easily through urine in the TUR cavity, there is a risk that the energy may extend beyond the intended treatment area. Therefore, extra caution is needed when targeting the peripheral zone near the rectum during HIDU, particularly in patients with prostate calculi.

## 3 Clinical Results of Whole‐Gland Therapy Using HIFU and HIDU

Long‐ and medium‐term clinical outcomes of HIFU for localized PC have been previously reported. Crouzet et al. studied transrectal HIFU with a median follow‐up period of 6.4 years (range, 0.2–13.9 years). Among the patients, 60% (*n* = 596) underwent HIFU once, 38% (*n* = 383) twice, and 2% (*n* = 23) thrice. Additionally, 392 patients were treated with neoadjuvant hormone therapy (NHT) before HIFU [9]. Biochemical recurrence (Phoenix ASRRO definition [26]) was observed in 205 (21.2%) patients [9]. The 5‐ and 8‐year biochemical‐free survival rates (BFSRs) for low‐, intermediate‐, and high‐risk patients were 86%, 78%, and 68% and 76%, 63%, and 57%, respectively [9]. The 8‐year BFSRs in patients with and without NHT were 70% and 66%, respectively (*p* = 0.992) [9], while the 10‐year overall survival and disease‐specific survival rates were 80% and 97%, respectively [9]. Grade 1 urinary incontinence occurred in 18.7% of the patients, grade 2/3 incontinence in 5.0%, acute urinary retention in 7.6%, urethral stricture in 9.0%, and rectourethral fistula in 0.4% [9].

Three‐year outcomes of a prospective multicenter phase I study of near‐whole‐gland therapy in HIDU have been reported [27]. Among the 30 patients studied, prostate specific antigen (PSA) levels decreased by 95% to a median nadir of 0.33 ng/mL (interquartile range: 0.1–0.4); follow‐up biopsies detected clinically significant PC (csPC) in 34% and any cancer in 59% of the patients, while seven patients required salvage treatment within 3 years, with no new severe adverse events reported [27].

## 4 Improvement of Treatment Outcomes With Intraoperative Compression of the Prostate

As a technical ingenuity for sufficient energy irradiation to the treatment area, transrectal compression was developed [28], which involves introducing an additional 80–160 mL of water into the transducer balloon to apply pressure on the prostate; the distance from the rectal surface to the ultrasound transducer was set parallel to 20–30 mm, depending on the prostate volume [10]. During treatment, the degassed water was manually adjusted to the starting position by a physician [10]. Compression of the prostate helps reduce intra‐procedural prostatic swelling [29] and decreases target displacement [28], thus increasing treatment accuracy. Hyperechoic changes in the B‐mode of TRUS images indicate effective PC treatment with HIFU [20, 30]. However, the diffuse appearance of hyperechoic changes is less likely in the treated area because of the cooling effect caused by blood flow and the long path of HIFU propagation. Prostate compression reduces vascular flow in the intra‐prostatic plexus and helps minimize the heat‐sink effect. Furthermore, the compressed tissue reduces the HIFU propagation path, resulting in a higher intensity at the focal site to promote cavitation [31]. Transrectal prostate compression is a distinctive feature of HIFU treatment utilizing the transrectal approach, representing a technical innovation that cannot be implemented with the transurethral approach.

Shoji et al. reported the clinical results of a single HIFU treatment with a median follow‐up of 5 years (range, 9–144 months) in patients with localized PC. The BFSRs of patients who underwent HIFU for localized PC in total, low‐ (*n* = 102), intermediate‐ (*n* = 240), and high‐risk (*n* = 86) groups were 68.4%, 80.4%, 65.6%, and 61.6%, respectively, according to the D'Amico classification [10]. Multivariate logistic regression analyses demonstrated that the compression method significantly reduced the risk of biochemical failure in all risk groups: low‐ (odds ratio [OR], 0.178; *p* = 0.030), intermediate‐ (OR, 0.291; *p* < 0.0001), and high‐risk (OR, 0.316; *p* = 0.049). Additionally, NHT was a significant factor in lowering the biochemical failure risk in high‐risk patients (OR, 0.225; *p* = 0.015) [10]. With regard to adverse events, transient urinary incontinence (3.7%), grade 2/3 urethral strictures (11.2%), erectile dysfunction (8.6%), and rectourethral fistulas (0.46%) were observed, with no significant differences in complications between patients treated with compression and those treated conventionally [10].

## 5 Clinical Results of Focal Therapy Using HIFU and HIDU

The development of multiparametric MRI has contributed to an accurate diagnosis of PC. Multi‐parametric MRI (mpMRI), which combines anatomical and functional evaluations of the prostate [32], is considered a useful modality for the detection of csPC [33]. CsPC is defined as PCs > 0.5 cm^3^ or tumor category ≥ T3 in whole‐mount specimens [34], and is regarded as a target to be managed to control PC progression [21, 35]. Reportedly, MRI‐TRUS fusion biopsy, combined with systematic biopsy, achieved a 90%–95% detection rate for index lesions, which are the largest or highest Gleason score tumors in patients with csPC [36, 37]. The development of mpMRI contributes to tailor‐made treatment, such as “focal therapy” that cures csPC while preserving the anatomical structures related to urinary and sexual functions [38, 39]. Focal therapy is indicated for patients with localized PC, including those with Gleason score 3 + 3 and an MRI‐visible lesion, and those with Gleason score 3 + 4 or higher; cases with Gleason score 4 + 4 should be considered cautiously, taking into account tumor size and patient age. It is not indicated for cases with Gleason score 4 + 5 or higher, or when extracapsular extension or seminal vesicle invasion is suspected [38]. Over the past decade, clinical results of focal therapy with HIFU for localized PC have been reported. Oncological outcomes were evaluated based on biochemical recurrence, pathological PC detection at follow‐up biopsy, and failure‐free survival (FFS), which is defined as the avoidance of local salvage therapy with surgery or radiotherapy, systemic therapy, metastases, or PC‐specific death [40]. Although the Phoenix ASTRO definition [26] has been used to evaluate biochemical recurrence after focal therapy, novel biomarkers are required [41, 42]. At present, follow‐up biopsies are generally performed to evaluate PC recurrence after focal therapy, depending on PSA elevation. However, the predictive values of the PSA nadir value [43], PSA density, percentage of PSA reduction [44], and MRI findings [21, 44] at the time of negative predictive value for PC detection on follow‐up biopsy have been reported.

Prospective clinical studies on focal therapy with HIFU and HIDU in the last 5 years have been reported, including at least 20 patients who were followed up for at least 12 months (Tables 1 and 2) [8, 12, 21, 23, 40, 45, 46, 47, 48, 49, 50, 51, 52, 53, 54]. With the development of HIFU technology in treatment planning, therapeutic technologies, and safety [7], ultrasound‐guided HIFU has become one of the most frequently used techniques in recent clinical studies on focal therapy for patients with localized PC [38]. Five‐year actuarial biochemical recurrence‐free survival rates in the low‐ and high‐risk groups were 75% and 36%, respectively [47]. In the follow‐up biopsy results of HIFU, the rates of csPC detection were 6.8% at 5 months [23], 8.9%–33% within 12 months [21, 45, 48, 49, 52, 54, 55], 12% at 24 months [12], 36.4% at 5 years [51], and 46% at 8 years [51] after treatment. In a large multicenter prospective study with medium‐term follow‐up, the FFS rates in low‐, intermediate‐, and high‐risk groups were 96%, 88%, and 84%, respectively, at 5 years of follow‐up [40] and 88%, 68%, and 65%, respectively, at 7 years of follow‐up [53], respectively. Further studies are required to evaluate the oncological role of focal therapy in localized PC. In previous studies, 3.8%–20% of patients who were assessed as having selection failure or recurrence received re‐treatment with HIFU after focal therapy with HIFU [40, 50, 55]. Regarding urinary function, continence was preserved in 80%–100% of patients after treatment [12, 21, 23, 40, 45, 46, 47, 48, 49, 50, 55]. In the oncological outcomes of MRI‐guided treatments (Table 2), csPC was detected at follow‐up biopsy in 6.8% [23], 12% [12], and 21% [8] of patients. Urinary incontinence rates ranged from 0% to 18%, and erectile dysfunction occurred in 4%–25% of cases, indicating that while functional outcomes are generally favorable, oncological control varies by modality and study. In the longitudinal analysis of urinary function during 24 months after treatment, the International Prostate Symptom Score (IPSS), IPSS QOL, overactive bladder symptom score, Expanded Prostate Cancer Index Composite (EPIC) urinary domain, and maximum urinary flow rate significantly deteriorated at 1 month after treatment but improved to preoperative levels at 3 or 6 months [55]. Shoji et al. focused on the win ratio as a statistical evaluation method that assesses not only oncological outcomes but also urinary incontinence [56]. In the win ratio analysis of short‐term clinical outcomes of focal therapy with HIFU and robot‐assisted radical prostatectomy (RARP) after propensity score matching, FFS was similar between focal therapy and RARP (*p* = 0.5044) over 36 months, whereas urinary function outcomes were significantly better in the focal therapy group (*p* < 0.0001) [56]. The win ratio of focal therapy versus RARP was 3.39, favoring focal therapy [56]. These findings suggest that focal therapy maintains oncological efficacy while preserving urinary function; however, long‐term clinical trials are needed to confirm its comprehensive benefits in patients with PC [56].

**TABLE 1:** Prospective clinical studies of focal therapy with transrectal ultrasound‐guided high‐intensity focused ultrasound that included at least 20 patients with follow‐up of at least 12 months.

Author (year)	Design	Diagnosis	Patients' number	Age	TNM classification (*n*)	PSA value, ng/mL (range)	Gleason score	Risk stratification (low, intermediate, high) (*n*)	Treatment range	Oncological outcomes	Functional outcomes	Median follow‐up (months)
Ahmed (2015)	Individual cohort study	MpMRI, biopsy (template mapping biopsy or TRUS‐guided biopsy)	56	63.9 (range, 51–76)	T1c 16, T2a 9, T2b 18, T2c 11, T3a 2	7.4 (5.6–9.5)	3 + 3 to 4 + 4	7, 47, 2	Hemi‐ablation, focal ablation	CsPC detection at follow‐up biopsy: 19.2%	Pad‐free continence: 92.3%, Leak‐free, pad‐free continence: 92.0%, ED: 23%	12
Feijoo (2015)	Individual cohort study	MpMRI, TRUS‐guided biopsy (at least 20 cores)	71	70.2 (SD 6.8)	NA	6.1 (1.6–15.5)	3 + 3, 3 + 4	NA	Hemi‐ablation	PC detection at follow‐up biopsy: 15%	Leak‐free, pad‐free continence: 100%	12
van Velthoven (2016)	Individual cohort study	MpMRI, TRUS‐guided biopsy	50	74 (IQR 70–77)	T1c 16, T2 34	6.3 (range 3.9–8.3)	3 + 3 to 4 + 3	24, 26, 0	Hemi‐ablation	5‐year actuarial Phoenix recurrence‐free survival in low‐ and intermediate‐risk groups: 75% and 36%	Pad‐free continence: 95%, ED 20%	35
Rischmann (2017)	Multicenter, cohort study	MpMRI, random biopsy (at least 12 cores) and target biopsy	111	64.9 (IQR 61–69)	T1 77, T2 33, unknown 1	5.6 (IQR 4.7–7.6)	3 + 3 to 4 + 3	NA	Hemi‐ablation	CsPC detection at follow‐up biopsy at 6–12 months after the treatment: 33%	Pad‐free continence: 97%, ED 22%	30.4
Guillaumier (2018)	Multicenter, cohort study	MpMRI, biopsy (template mapping biopsy or TRUS‐guided biopsy)	625	65 (IQR 61–71)	T1 65, T2a 82, T2b 73, T2c 93, Missing T2 subclassification 184, T3a 75, T3b 7	7.2 (IQR 5.2–10.0)	3 + 3 to ≥ 4 + 4	78, 316, 189 (missing data 16)	Wide and normal hemi‐ablation, focal ablation	Failure‐free survival in low‐, intermediate‐, high‐risk groups, total (5 years) 96%, 88%, 84%, and 88%	Leak‐free, pad‐free continence: 80%	56
Ganzer (2018)	Multicenter, cohort study	TRUS‐guided (12 cores) systematic biopsy	49	63.4 (SD 8.3)	NA	6.2 (SD 2.1)	3 + 3, 3 + 4	NA	Hemi‐ablation	CsPC detection at follow‐up biopsy on treated side 8.2%, rates of csPC detection at follow‐up biopsy on contralateral side 2.0%	Leak‐free, pad‐free continence: 100% ED: 30%	12
Johnston (2019)	Individual cohort study	MpMRI, prostate biopsy	107	66 (range 47–81)	T1 9, T2 90, T3 8	7.7 (range 1.2–26.2)	3 + 3 to 4 + 4	13 (low), 94 (intermediate and high)	Hemi‐ablation, focal ablation	PC detection at follow‐up biopsy 73%	Leak‐free, pad‐free continence: 99% ED: 14%	30
Stabile (2019)	Multicenter, cohort study	MpMRI, biopsy (template mapping biopsy or TRUS‐guided biopsy)	1032	65 (range 60–70)	T1 78, T2 802, T3 123	7 (IQR 4.9–9.7)	3 + 3 to 4 + 4	NA	Hemi‐ablation, focal ablation	CsPC detection at follow‐up biopsy: 36.4% (at 5 years), 46% (at 8 years)	NA	36
Shoji (2020)	Individual cohort study	MpMRI‐TRUS fusion image‐guided biopsy	90	70 (range 39–85)	T2a 71, T2b 15, T2c 4	7.26 (range 2.48–19.95)	3 + 3 to 4 + 4	31, 44, 15	Hemi‐ablation, focal ablation	CsPC detection at follow‐up biopsy 8.9% at 6 months after the treatment	Leak‐free, pad‐free continence: 100% ED: 14%	21
Abreu (2020)	Individual cohort	MpMRI‐TRUS fusion image‐guided biopsy	100	65 (IQR 59–70)	T1c 85, T2a 12, T2b 1, T2c 2	5.9 (IQR 4.5–7.2)	3 + 3 to 4 + 4	28, 67, 5	Hemi‐ablation	CsPC detection at follow‐up biopsy 31%	NA	20
Reddy (2022)	Multicenter, cohort study	MpMRI, biopsy (template mapping biopsy or TRUS‐guided biopsy)	1379	66 (range 60–71)	T1 95, T2a 276, T2b 140, T2c 209, missing T2 subclassification 398, T3a/b 151, missing data 110 (8.0)	6.9 (range 4.9–9.4)	3 + 3 to ≥ 8	84, 896, 386 (missing data 13, Gleason 3 + 3 = 6, maximum cancer core length < 6 mm, rT1 20)	Quadrant, hemi‐ablation, hockey‐stick	Failure‐free survival in low‐, intermediate‐, high‐risk groups, total (7 years) 88%, 68%, and 65%	NA	32
Westhoff (2023)	Individual cohort	MpMRI‐TRUS fusion image‐guided biopsy	50	68 (range 48–80)	T1c 50	6.5 (range 1.2–9.9)	3 + 3, 3 + 4	35, 15, 0 (Cancer of the Prostate Risk Assessment)	Focal ablation	CsPC detection at follow‐up biopsy 26% at 12 months	Urinary continence was not impaired. IIEF score changed from a median of 20 points to 13 points	42
Shoji (2024)	Individual cohort study	MpMRI‐TRUS fusion image‐guided biopsy	240	Low‐risk group: 67 (40–79), Intermediate risk group: 69 (39–81), High‐risk group: 71 (48–85)	T2a 159, T2b 21, T2c 60	Low‐risk group: 6.25 (2.48–9.35), Intermediate risk group: 6.45 (2.24–20.0), High‐risk group: 6.61 (3.17–19.95)	3 + 3 to 4 + 4	51, 107, 82	Region targeted focal ablation	Phoenix ASTRO recurrence‐free survival in low‐, intermediate‐, and high risk group: 92.2%, 89.7%, and 85.4%	Leak‐free, pad‐free continence: 100%	Low‐risk group: 54, Intermediate risk group: 48, High‐risk group: 48

Abbreviations: ED = erectile dysfunction, IQR = interquartile range, MpMRI = multi‐parametric magnetic resonance imaging, NA = not available, TRUS = transrectal ultrasound.

**TABLE 2:** Prospective clinical studies of focal therapy with magnetic resonance imaging‐guided ultrasound treatment that included at least 20 patients with follow‐up of at least 12 months.

Author (year)	Design	Treatment modality	Diagnosis	Patients' number	Age	TNM classification (*n*)	PSA value, ng/mL (range)	Gleason score	Risk stratification (low, intermediate, high) (*n*)	Treatment range	Oncological outcomes	Functional outcomes	Median follow‐up (months)
Ghai (2021)	Individual cohort study	HIFU	MRI‐TRUS fusion image‐guided biopsy	44	67 (IQR 62–70)	NA	6.4 (IQR 4.3–9.6)	3 + 4, 4 + 3	NA	Focal ablation	CsPC detection at follow‐up biopsy: 6.8% at 5 months	Urinary incontinence 0%, ED: 4%	24
Ehdaie (2022)	Multicenter, cohort study	HIFU	MRI‐targeted and systematic biopsy	101	63 (IQR 58–67)	≤ T1c 84	5.7 (IQR 4.2–7.5)	3 + 4, 4 + 3	NA	Focal ablation	CsPC detection at follow‐up biopsy: 12%	Urinary incontinence: 18% (CTCAE grade 1, 2), ED: 20% (CTCAE grade 1, 2)	24
Klotz (2021)	Multicenter, cohort study	HIDU	MpMRI, prostate biopsy	115	65.0 (range 46–79)	T1c 89, T2a 20, T2b 1, T2, unspecified substage	6.3 (range 4.6–7.9)	3 + 3 to 4 + 3	38, 77, 0	Urethra‐sparing ablation	CsPC detection at follow‐up biopsy in the patients: 21%	Urinary incontinence less than 1%, ED: 25%	12

Abbreviations: CTCAE = Common Terminology Criteria for Adverse Events version 4.03, ED = erectile dysfunction, HIDU = high‐intensity directional ultrasound, HIFU = high‐intensity focused ultrasound, IQR = interquartile range, MRI = magnetic resonance imaging, NA = not available, TRUS = transrectal ultrasound.

Transient deterioration in urinary function was thought to be due to transient prostatic swelling that occurred immediately after treatment [29]. In the analysis of the risk of transient deterioration of urinary function, a lower maximum pretreatment flow rate (OR, 1.083; *p* = 0.023) and treatment of the anterior portion of the transition zone (OR, 3.386; *p* = 0.029) were significant risk factors for deterioration, with ≥ 32% of the preoperative status of maximum flow rates in the multivariate logistic regression analysis [57]. Erectile dysfunction occurs in 14%–30% of patients [12, 21, 23, 45, 47, 48, 49, 50]. In a longitudinal analysis of the International Index of Erectile Function‐5 (IIEF‐5), which evaluates erectile function, IIEF‐5 was significantly impaired in the initial 3 months after treatment compared with the pretreatment values; however, it improved to baseline at 6 months after focal therapy [21], In a recent study, lower pre‐procedural IIEF‐5 score (OR, 0.812; *p* = 0.005), lower pre‐procedural score of the sexual domain of the EPIC (OR, 0.960; *p* = 0.038), and treatment of the edge of the peripheral zone in proximity to the neurovascular bundle (treated vs. untreated: OR, 8.048; *p* = 0.028) were significant risk factors for severe erectile dysfunction 12 months after treatment in multivariable logistic regression analysis [58]. These results will contribute to informed consent of patients at risk of developing severe erectile dysfunction after treatment. The clinical results of focal therapy with HIDU have been reported as urethra‐sparing treatment after 12 months of follow‐up in a multicenter study [8]. After the treatment of patients with low‐ and intermediate‐risk PC, the csPC detection rate was 21% in the follow‐up biopsy. Regarding functional outcomes, the rates of urinary incontinence and erectile dysfunction were < 1% and < 25%, respectively [8].

Although clinical outcomes of ultrasound‐based therapy have been reported, this treatment differs fundamentally from methods that induce tissue destruction through resection or thermal ablation at the tip of a puncturing device. Instead, it transmits focused ultrasound energy to treat the targeted region noninvasively. This approach offers several advantages, including the ability to treat without making incisions or punctures and with greater flexibility in targeting. However, it also requires precise intraoperative adjustments, such as the use of compression methods and adaptation to real‐time changes in prostate anatomy. Moving forward, promoting education and dissemination of these technical skills will be essential to reducing variability in clinical outcomes across institutions.

## 6 Current Insurance Coverage Status of HIFU and HIDU

HIFU for PC is covered by public health insurance in France. In the United Kingdom, the National Institute for Health and Care Excellence allows its use under specific conditions when conducted as part of a clinical study. In the United States, HIFU has been approved for the ablation of prostate tissue. In Japan, it was approved as an advanced medical technology in 2023 and is currently being implemented as part of a multi‐institutional clinical study. HIDU is partially supported by public insurance in limited institutions in countries such as Canada, Switzerland, and Germany. In the United States, it has been approved as a device for prostate tissue ablation. In Japan, HIDU remains an unapproved medical device and is not covered by public insurance. It should be initiated as a specified clinical study without further delay.

## 7 Possibilities of HIFU and Low‐Intensity Focused Ultrasound as Immunomodulatory Cancer Therapies

HIFU [59, 60] and low‐intensity focused ultrasound (LOFU) [61] have emerged as promising therapeutic modalities for PC, not only for their direct cytotoxic effects but also for their ability to modulate the immune system. Additionally, HIFU ablates tumors and enhances anti‐tumor immunity by inducing immunogenic cell death, releasing tumor‐associated antigens and damage‐associated molecular patterns, and promoting CD8^+^ T cell activation [59]. HIFU reshapes the tumor microenvironment, reducing the number of immunosuppressive cells such as Tregs and myeloid‐derived suppressor cells [59]. Another study showed increased CD4^+^ and CD8^+^ T cell infiltration and an enhanced immune response [60]. LOFU is a non‐invasive immune‐priming therapy that enhances tumor immunogenicity by inducing heat shock protein expression and promoting antigen presentation to CD8^+^ T cells [61]. In PC, the combination of LOFU and ablative radiation therapy significantly improved tumor clearance in immunocompetent mice, demonstrating its potential to convert focal tumor ablation into systemic anti‐tumor immunity [61]. This approach may synergize with immunotherapy, offering a promising adjuvant strategy for advanced PC treatment [61].

## 8 Future Perspectives on Focal Therapy and Ultrasound‐Based Immunomodulation for PC

As a standard treatment strategy for localized PC, focal therapy should demonstrate superior treatment outcomes compared with conventional therapies in selected patients. To verify its effectiveness, it is essential to conduct pair‐matched and historically controlled studies that compare focal therapy with radical treatment. However, designing a randomized controlled trial is challenging because of the different characteristics of patients undergoing focal and radical treatments. In Japan, a multi‐institutional prospective study on focal therapy using HIFU has been approved as an advanced medical technology by the Minister of Health, Labor, and Welfare. This study compared oncological and functional outcomes of HIFU‐based focal therapy and radical prostatectomy in pair‐matched patients, contributing to the evaluation of the effectiveness of HIFU in treating localized PC. By leveraging the characteristics of ultrasound, HIFU and HIDU can be appropriately applied to enable focal therapy for tumors located anywhere within the prostate. However, HIDU presents challenges, such as a lack of established treatment strategies for cases with prostatic calculi, insufficient long‐term clinical data involving large patient cohorts, and high costs associated with disposable HIDU devices. Additionally, compatibility with specific MRI systems remains a barrier to broader adoption. The high cost is partly attributable to the need to occupy the MRI scanner for several hours per treatment. Moreover, when attempting to introduce HIDU using an existing MRI suite, additional construction work is often required, such as drilling holes to pass cables for operating the system from outside the MRI room.

Moving forward, new treatment strategies utilizing the immune responses induced by HIFU and LOFU are anticipated. Given that PC is traditionally considered immunologically cold, applying HIFU and LOFU to stimulate anti‐tumor immune responses could be beneficial not only for localized PC but also as a potential treatment strategy for advanced PC.

## 9 Conclusion

HIFU is a widely used focal therapy modality for localized PC, supported by long‐term oncological and functional outcomes. HIDU remains in the early stages of clinical adoption, with only limited short‐term data available, and still faces challenges related to cost and treatment optimization. Both HIFU and LOFU exhibit immunostimulatory effects, thus opening new therapeutic possibilities. Future studies should continue to evaluate the long‐term efficacy of HIFU while further investigating the feasibility of HIDU and addressing its limitations. Additionally, the potential synergy among HIFU, LOFU, and immunotherapy warrants further exploration for both localized and advanced PC treatments.

## Author Contributions

**Sunao Shoji:** conceptualization, data curation, funding acquisition, methodology, supervision, writing – original draft, writing – review and editing. **Kumpei Takahashi:** data curation, investigation. **Jun Naruse:** data curation, writing – original draft. **Yoshiaki Kawamura:** supervision, writing – review and editing.

## Ethics Statement

The authors have nothing to report.

## Consent

The authors have nothing to report.

## Conflicts of Interest

Sunao Shoji is an Editorial Board member of International Journal of Urology and a co‐author of this article. To minimize bias, they were excluded from all editorial decision‐making related to the acceptance of this article for publication.
